# Severe polyserositis induced by the 13-valent pneumococcal conjugate vaccine: a case report

**DOI:** 10.1186/s13256-017-1305-4

**Published:** 2017-05-20

**Authors:** Pierre Tawfik, Elie Gertner, Charlene E. McEvoy

**Affiliations:** Regions Hospital, 640 Jackson St, St Paul, MN 55101 USA

**Keywords:** Pneumococcal conjugate vaccine, Serositis, Pleural effusion, Pericardial effusion, Case report

## Abstract

**Background:**

The United States Advisory Committee on Immunization Practices recommends administration of the 13-valent pneumococcal conjugate vaccine in series with the 23-valent pneumococcal polysaccharide vaccine for prevention of pneumonia in the elderly. Reports of autoimmune or auto-inflammatory diseases as a result of pneumococcal vaccination, especially pneumococcal conjugate vaccine, are extremely rare.

**Case presentation:**

We present a case of severe serositis in a 75-year-old Caucasian woman complicated by pericardial and pleural effusions in the setting of recent 13-valent pneumococcal conjugate vaccine vaccination and no other obvious etiology. Our patient required steroid treatment, thoracentesis, chest tube, and pericardial window and subsequently recovered to her baseline.

**Conclusions:**

To the best of our knowledge, no such reaction to the 13-valent pneumococcal conjugate vaccine has previously been documented. Although the benefits of vaccination outweigh the risks, knowledge of this potential side effect can help clinicians in diagnosis and treatment of similar patients.

## Background

In August, 2014 the United States Advisory Committee on Immunization Practices recommended vaccination of adults aged ≥65 years with the 13-valent pneumococcal conjugate vaccine (PCV13) [1]. The PCV13 vaccine is now routinely given in series with the 23-valent pneumococcal polysaccharide vaccine (PPSV23) for prevention of community-acquired pneumonia in the elderly. The incidence of autoimmune or auto-inflammatory diseases as a result of pneumococcal vaccination is extremely rare [2]. Borella *et al.* identified only 14 case reports of possible rheumatic disorders after pneumococcal vaccines from 1980 to 2013. All 14 cases were linked to PPSV23 [3]. Also, a recently described syndrome, the “autoimmune/auto-inflammatory syndrome induced by adjuvants” (ASIA), proposes that post-vaccination phenomena may be due to reactions to adjuvants within vaccines [4].

We report a case of severe serositis with elevated markers of inflammation requiring medical and surgical intervention in a patient with recent pneumococcal conjugate vaccine (PCV13) vaccination and no other obvious etiology. The PCV13 vaccine is an adjuvanted vaccine, containing aluminum, while PPV23 is nonadjuvanted. The NOD-like receptor protein (NLRP3) inflammasome plays a crucial role in the immunostimulatory property of aluminum. However, activation of the NLPR3 inflammasome pathway is also critically involved in the development of autoimmune and inflammatory diseases [5]. This appears to be the first case report of systemic inflammation secondary to PCV13 in a patient without previous underlying autoimmune disease.

## Case presentation

A 75-year-old Caucasian woman with past medical history of atrial fibrillation, hypertension, and remotely treated breast cancer presented with dyspnea, non-productive cough, subjective fevers, polyarthralgias, myalgias, and consequent sleep disturbance of 10 days’ duration. Her medications were hydrochlorothiazide, losartan and simvastatin. Eighteen days earlier she had been seen for a routine health-care visit and received PCV13. Soon thereafter she developed a large, erythematous, warm local reaction to the vaccine, which was still present. Her vital signs included a pulse rate of 126 beats/minute, blood pressure of 99/63 mmHg, a maximum temperature of 36.6 degrees Celsius, and oxygen saturation of 83% on room air requiring bilevel positive airway pressure therapy. She had reduced breath sounds in the left lung field. Her white blood cell (WBC) count was 19.1 (normal 4–11 K/uL), creatinine level was 1.5 (normal 0.52–1.04 mg/dL), and B-type natriuretic peptide level was 1670 (normal 0–900 pg/mL). All blood bacterial cultures, urine streptococcus pneumonia and legionella antigens, mycoplasma titers, and Lyme antibody test results were negative. A chest X-ray and computed tomography (CT) scan showed a large left pleural effusion and a large pericardial effusion. An echocardiogram confirmed the pericardial effusion. Her erythrocyte sedimentation rate (ESR) was 59 (normal 0–20 mm/hour), C-reactive protein (CRP) level was 62.7 (normal 0–0.9 mg/dL), and her rheumatoid factor was 15 (normal 0–11 IU/mL). Her antinuclear antibody test result was negative. Test results for anti-Sm, -double stranded deoxyribonucleic acid (dsDNA), -histone, -cardiolipin, -ribonucleoprotein, -centromere, -topoisomerase I, -Ro, -La, -thyroid, -neutrophil cytoplasmic (-myeloperoxidase and -proteinase 3) antibodies were all negative. Her complement levels were normal.

Thoracentesis and pericardial window were required. Pleural fluid appeared bloody and was exudative by Light’s criteria (serum lactate dehydrogenase [LDH] 765 U/L, serum total protein 7.7 g/dL; pleural LDH 749 U/L, red blood cells 320,000/uL, nucleated cells 3368/uL [PMN 62%, lymphocytes 22%, macrophages/histiocytes 14%, mesothelial cells 2%], glucose 97 mg/dL) [6]. Cytology test results of the pleural fluid were negative. The pericardial fluid was exudative and hemorrhagic and cultures and cytology test results were negative. A pericardial biopsy showed organizing effusion containing hemorrhage and acute inflammation with reactive changes and mesothelial hyperplasia. Cultures of the pericardial biopsy were negative.

The pleural fluid re-accumulated so a chest tube was placed. Repeat fluid analysis was similar and cultures were negative. Given the severity of her illness, her serositis was treated with corticosteroids rather than nonsteroidal anti-inflammatory medications [7]. She received 40 mg of prednisone daily for 2 weeks with a good initial response and tapered off steroids over the next 3 months. Her vital signs recovered to normal range throughout the admission. The chest tube and pericardial drain were removed prior to discharge. A follow-up chest X-ray one year after discharge revealed no recurring pleural effusions. She has remained asymptomatic now for one and a half years since discharge.

## Discussion

This case demonstrates a rare adverse effect likely related to PCV13 vaccination. Our patient had a severe focal skin reaction to the vaccine and progressively developed polyarthralgias, myalgias, and finally marked serositis of the pleura and pericardium. The temporal association, the absence of identifiable infective or neoplastic etiologies, and improvement with steroids suggests a reaction to the vaccine. Thus, to our knowledge this report documents the first systematic inflammatory reaction to PCV13 in a patient without previous autoimmune disease.

One limitation of this report is the possibility of falsely negative microbial cultures or cytology test results. This is unlikely since the pleural fluid was analyzed twice. Another limitation is the possibility of viral etiology. Viruses such as echovirus and coxsackievirus are known causes of pericarditis and steroids can be used for treatment [8]. Arguing against this is the temporal association to the PCV13 vaccine, the marked local reaction, and the fact that viruses are not common causes of pleural effusions [9].

We initially considered a recently proposed syndrome called “autoimmune/auto-inflammatory syndrome induced by adjuvants” (ASIA) as the etiology but did not feel the patient met all the necessary criteria. Shoenfeld and Agmon-Levin proposed this syndrome wherein an adjuvant, such as the aluminum in PCV13, can trigger an autoimmune/auto-inflammatory phenomenon [4]. Our patient met the ASIA criteria of exposure to an external stimulus, improvement with removal of the inciting agent, and appearance of typical symptoms including myalgias, arthralgias, and sleep disturbance. However, she did not have the neurologic or cognitive changes noted in the syndrome. Regardless, the adjuvant in PCV13 may have played a strong role in the systemic inflammation in our patient.

## Conclusions

In summary, while immunization with PCV13 is highly recommended, rarely an auto-inflammatory response may occur. Recognition of this potential adverse effect can enable more thorough questioning regarding prior-to-presentation vaccines. It may also facilitate prompt administration of steroids after alternate etiologies of inflammation have been ruled out.

## Abbreviations

ASIA: Autoimmune/auto-inflammatory syndrome induced by adjuvants
dsDNA: double stranded deoxyribonucleic acid
NLRP: NOD-like receptor protein
PCV13: Pneumococcal conjugate vaccine
PPSV23: Pneumococcal polysaccharide vaccine
